# The wolf in sheep’s clothing: vasovagal syncope in acute aortic dissection

**DOI:** 10.1186/s12245-024-00664-8

**Published:** 2024-07-02

**Authors:** Viviane Donner, Hadrien Beuret, Simon Savoy, Vincent Ribordy, Christine D. Sadeghi

**Affiliations:** 1 Department of Emergency Medicine, Fribourg Cantonal Hospital, Ch. des Pensionnats 2-6, Fribourg, CH 1700 Switzerland; 2 Division of Intensive Care, Department of Anaesthesiology, Clinical Pharmacology, Intensive Care and Emergency Medicine, Geneva University Hospital, Rue Gabrielle-Perret-Gentil 4, Geneva, CH 1211 Switzerland; 3 Department of Cardiology, Fribourg Cantonal Hospital, Ch. des Pensionnats 2-6, Fribourg, CH 1700 Switzerland; 4 Department of Intensive Care Medicine, Fribourg Cantonal Hospital, Ch. des Pensionnats 2-6, Fribourg, CH 1700 Switzerland; 5 Faculty of Medicine, University of Fribourg, Fribourg, Switzerland

**Keywords:** Acute aortic dissection, Vasovagal syncope, Painless aortic dissection, Aortic depressor nerve, Risk stratification, Aortic arch baroreflex

## Abstract

**Background:**

The presentation of acute aortic dissection can pose a challenge for emergency physicians, as it may occur without pain. Atypical presentations can lead to significant delays in diagnosis and increased mortality rates.

**Case description:**

Our case illustrates that isolated painless syncope can be a rare presenting symptom of acute aortic dissection type A. What is unique about our case is the limited extension of the dissection tear and the availability of Holter monitoring during the syncopal episode.

**Conclusion:**

This constellation provides insight into the pathophysiological mechanism of the syncope in this patient. Mechanisms of syncope related to acute aortic dissection are diverse. We show that vasovagal activation not related to pain can be the underlying mechanism of syncope in acute aortic dissection type A. Although excessive vasovagal tone in the setting of aortic dissection has been hypothesized in the past, it has never been as clearly illustrated as in the present case. This also highlights the challenge in risk stratification of syncope in the emergency department.

## Background

Acute aortic dissection (AAD) classically presents with severe or worst pain ever (in about 93% of cases) [1]. Atypical AAD presentation, without chest pain, is uncommon and can be challenging for the emergency physician. This can result in significant delay in diagnosis and an increased mortality [2–5]. Factors associated with delay in diagnosis are female patients, painless presentation, absence of pulse deficit or hypotension [4].

Syncope is an uncommon symptom of AAD, reported in up to 19% of AAD type A, while it affects only 3% of patients with type B dissections [1]. It can occur in isolation, without any chest or back pain symptoms, in up to 3% of AAD [6]. According to a retrospective series, bradyarrythmias are common in AAD type A, sinus bradycardia occurring in up to 11% of cases and AV block in up to 1.9% [7].

## Case presentation

An 82-year-old female patient collapsed at home on her way to the restroom. Her husband heard the fall, found her drowsy but responsive, and alerted the Emergency Medical Service (EMS). On EMS arrival, the patient was found lying in the corridor. Her Glasgow Coma Scale (GCS) was 14 (4/4/6), and her vitals were noticeable for bradycardia at 45 bpm and mild hypotension (BP 95/55 mmHg). She was treated with 1 mg of atropine with resolution of both bradycardia and hypotension.

On emergency department (ED) admission, the patient had no recall of her collapse. She complained of mild nausea, but denied any chest pain, back pain, dyspnea, or headache. Her medical history was relevant for well-controlled arterial hypertension on a longstanding treatment of Metoprolol and Irbesartan, but no previous history of transient loss of consciousness. The clinical examination was characterised by sinus rhythm (73 bpm), blood pressure which had dropped back into the low range (94/64mmHg) and slight drowsiness. The peripheral pulses were palpable. Blood pressure was symmetrical in both arms. The cardiac and respiratory auscultation did not reveal a murmur or abnormal sounds. Carotid auscultation was normal. Her jugular veins were not distended. Her neurological assessment did not show any focal signs. The laboratory workup was unremarkable and admission EKG showed discreet changes compatible with hypertension.

Syncope work-up included supine and standing blood pressure measurements and a carotid sinus massage, which were both negative. A recent outpatient echocardiography showed a normal left ventricular function (LVEF 65%) without significant valve disease nor pulmonary hypertension. During her syncope and upon evaluation in the emergency room, the patient was wearing a Holter, as part of an ongoing workup for chronic dizziness with more than two years of evolution. At the time of admission, the Holter data could not be retrieved. The patient was hospitalised for continuous cardiac monitoring.

**Fig. 1:**
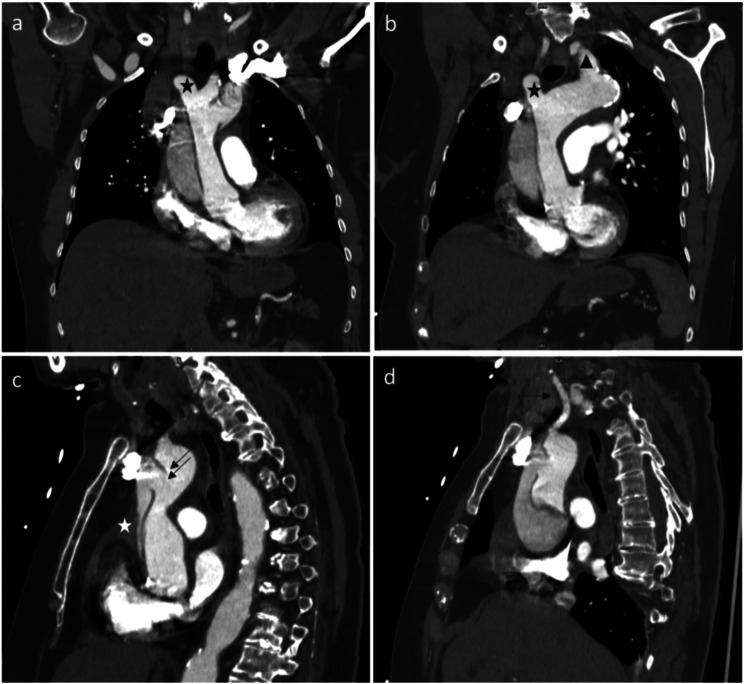
AngioCT of the chest showing a type A aortic dissection sparing the supraaortic vessels. **(a)** sparing of the brachiocephalic trunk (black star) **(b)** sparing of the brachiocephalic trunk and the left subclavicular artery (black triangle) **(c)** the anterior aortic tear (double black arrows) spreading proximally toward the aortic sinus, and pericardial effusion (white star). **(d)** the aortic tear sparing the left common carotid artery (single black arrow)

During her monitoring, the patient was asymptomatic and did not have any recurrent episode of bradycardia. Her blood pressure remained in a slightly low range for her age (SBPs 100–110 mmHg) up to 12 h after her collapse and then normalised. An echocardiography was performed on day 2 and revealed a pericardial effusion of 8–10 mm without sign of tamponade. The work up was completed with a thoracic computed tomography angiography. This revealed an acute aortic dissection type A according to the Stanford classification, or type A E1 M0 according to Sievers [8]. The primary entry of the dissection tear was located in the ascending aorta just above the aortic sinus extending through to the aortic arch, stopping before the left carotid artery, sparing the coronary arteries and the supra-aortic trunks (Fig. 1). She was referred to a tertiary university hospital where she underwent ascending aorta replacement. The postoperative course was uneventful. She was discharged from the tertiary hospital on day 14 for cardiac rehabilitation.

**Fig. 2:**
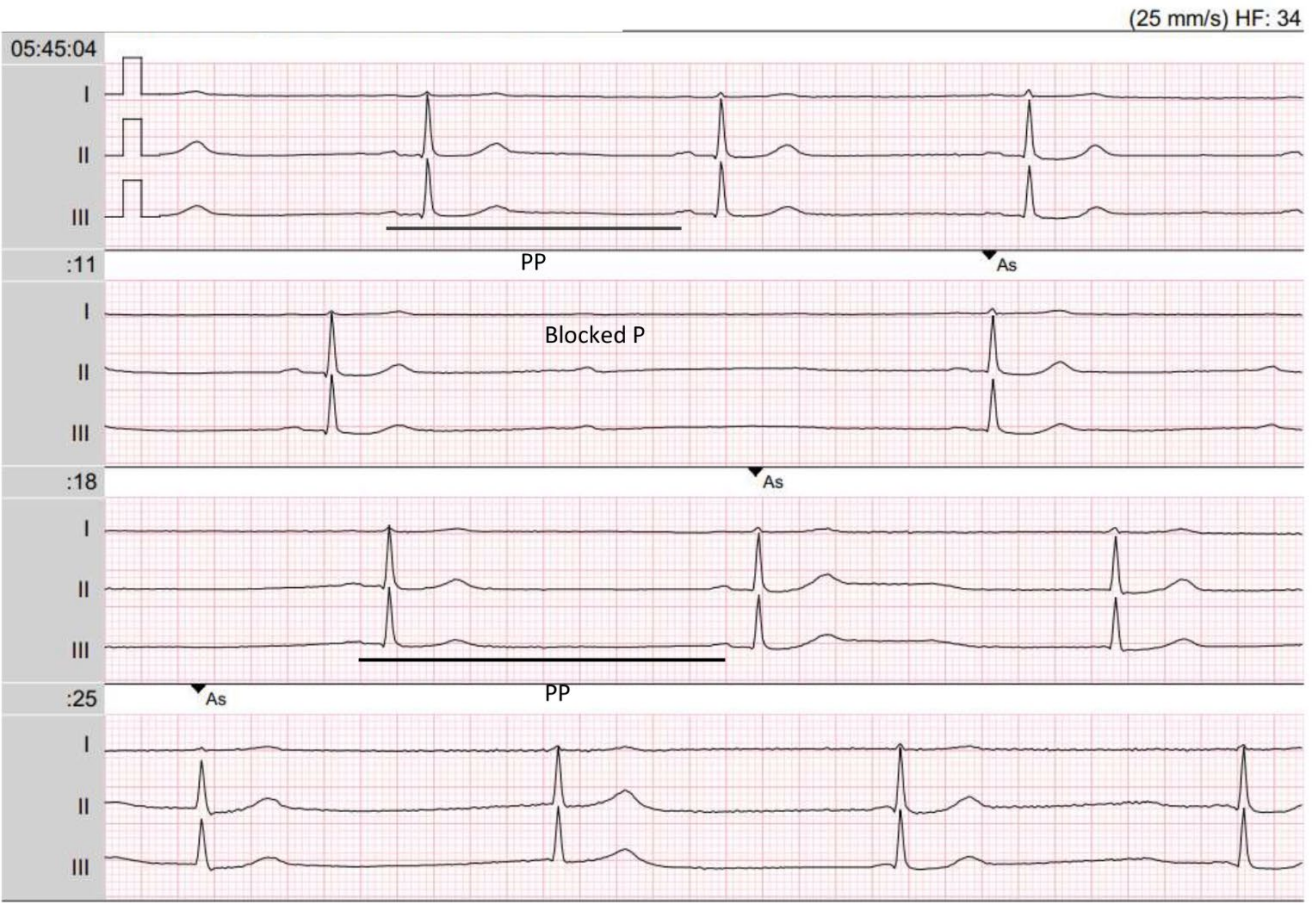
This Holter monitoring was recorded at the time of the collapse, two minutes before EMS activation. The Holter shows sinus bradycardia, progressive lengthening of PP interval, followed by 2nd grade AV-Block, sinus node arrest and finally junctional escape rhythm. This pattern is pathognomonic of vagal activation

Figure 2 shows the Holter monitoring that the patient was wearing during her syncope and that we retrospectively obtained. The monitoring shows sinus bradycardia, progressive lengthening of PP interval, followed by 2nd degree AV block, sinus node arrest and finally junctional escape rhythm. The observed sequence of different rhythms on the Holter monitor suggests a pronounced activation of the parasympathetic nervous system.

## Discussion

Mechanisms of syncope in the setting of AAD may vary from case to case but are commonly thought to be due to cardiac tamponade, coronary involvement or cerebral ischemia secondary to precerebral vessel involvement [9, 10]. In one study however, approximately half of patients presenting with an AAD and a syncope were not found to have any of the classical mechanical complications explaining the syncope [6]. Increasing vagal tone is another possible mechanism explaining syncope and bradyarrythmia in the setting of AAD [6, 10] and our case is an illustration of this mechanism.

Vasovagal syncope (or reflex syncope) is a common neurologically mediated form of syncope typically associated with a precipitating factor such as pain or stressors. The physiopathology of vasovagal syncope has not yet been entirely elucidated but involves autonomic pathways: hypotension through vasodepression due to sympathetic system inhibition, and bradycardia through parasympathetic-mediated cardioinhibition [11]. Vasovagal mechanisms have been suggested to be involved in type A AAD related syncope in the past [6, 10]. Dissection tear can stimulate mechanoreceptors located in the vascular wall of the aortic arch, triggering the aortic arch baroreflex via an afferent branch of the vagal nerve called the aortic depressor nerve, which provides sensory innervation of the aortic arch [12, 13]. Vagal-mediated efferences can then induce hypotension, as well as cardioinhibition slowing the sinus and AV node conduction. This can result in sinus bradycardia, sinus arrest, functional AV block, and sometimes asystole which can be reverted by atropine [14].

In our patient, the dissection tear showed a unique limited extension with precerebral and coronary vessel sparing. The absence of tamponade physiology or valvular dysfunction, the documentation of a functional atrio-ventricular block, the adequate response to atropine, and the negative syncope work up support the hypothesis of a vagally mediated syncope, through activation of the aortic arch baroreflex. The other symptoms of the patient such as borderline low blood pressure, slight decrease of consciousness and nausea can also be explained via persistently increased vagal tone [12].

Syncope is a non-specific symptom that can be due to multiple causes, ranging from the benign to the life-threatening. Risk stratification is an important part of the evaluation of syncope in the ED. International guidelines recommend managing patients with presumptive vasovagal syncope in an outpatient setting, as this category of syncope is generally regarded as low-risk and distinct from syncope of cardiac origin [15, 16]. Scores, such as the San Francisco rule, provide guidance for syncope risk stratification but have not been shown to be better than clinical judgment in identifying patients at higher risk of adverse outcomes [17]. A more recent externally validated syncope score, the Canadian Syncope risk score (CSRS), would categorize this patient as low risk if cardiac syncope was not suspected [18]. Considering a recent normal echocardiography, a benign EKG in the ED and no history of coronary heart disease, cardiac syncope was unlikely. However, some features of this case were concerning. A new onset syncope in an elderly patient, a fall, a non-specified prehospital rhythm disturbance and borderline blood pressure in the ED were considered enough arguments for inpatient rhythm and clinical monitoring, as a cardiac cause of syncope could not be formally ruled out.

This raises the question if syncope of unclear origin should trigger further testing in the ED, such as biomarkers. There is a debate surrounding biomarker testing to rule out pulmonary embolism in patients presenting to the ED with syncope of unclear origin. It has recently been shown that the prevalence of pulmonary embolism in patients presenting to the ED with syncope is very low [19, 20] and therefore indiscriminate biomarker testing should be discouraged because this strategy would result in low imaging yield and high costs [21]. Similarly, D-dimer levels may assist in the diagnosis of AAD [22]. The prevalence of AAD in patients presenting to the ED with syncope is not well known but is likely extremely low as suggested by a recent observational study including patients presenting with potential AAD symptoms [23]. By analogy to pulmonary embolism, syncope, which is an uncommon presentation of AAD, should not prompt indiscriminate D-dimer testing in the ED [24]. The balance between rapidly identifying life-threatening conditions and preventing harm from over-investigation [25] remains a delicate challenge in the fast-paced, time-critical environment of the emergency department.

In conclusion, this case serves as a reminder that AAD should be considered in the differential diagnosis of patients presenting to the emergency department with syncope. It also demonstrates that AAD can manifest specifically as vasovagal syncope. To the best of our knowledge, isolated painless vasovagal syncope in the setting of AAD has never been reported and is probably a rare presentation. Finally, despite many clinical tools, risk stratification of syncope in the ED remains challenging and still relies on clinical gestalt.

## Data Availability

No datasets were generated or analysed during the current study.
